# Dual Transcriptomics of Host-Pathogen Interaction of Cystic Fibrosis Isolate *Pseudomonas aeruginosa* PASS1 With Zebrafish

**DOI:** 10.3389/fcimb.2018.00406

**Published:** 2018-11-22

**Authors:** Sheemal S. Kumar, Julia I. Tandberg, Anahit Penesyan, Liam D. H. Elbourne, Nadia Suarez-Bosche, Emily Don, Eline Skadberg, Federico Fenaroli, Nicholas Cole, Hanne Cecilie Winther-Larsen, Ian T. Paulsen

**Affiliations:** ^1^Department of Molecular Sciences, Faculty of Science and Engineering, Macquarie University, Sydney, NSW, Australia; ^2^Department of Pharmaceutical Biosciences, Centre of Integrative Microbial Evolution, School of Pharmacy, University of Oslo, Oslo, Norway; ^3^Microscopy Unit, Faculty of Science and Engineering, Macquarie University, Sydney, NSW, Australia; ^4^Department of Biomedical Sciences, Faculty of Medicine and Health Sciences, Macquarie University, Sydney, NSW, Australia; ^5^Department of Biosciences, The Faculty of Mathematic and Natural Sciences, University of Oslo, Oslo, Norway

**Keywords:** RNA-Seq, host-pathogen interactions, virulence, *Pseudomonas aeruginosa*, zebrafish, innate immunity

## Abstract

*Pseudomonas aeruginosa* is a significant cause of mortality in patients with cystic fibrosis (CF). To explore the interaction of the CF isolate *P. aeruginosa* PASS1 with the innate immune response, we have used *Danio rerio* (zebrafish) as an infection model. Confocal laser scanning microscopy (CLSM) enabled visualization of direct interactions between zebrafish macrophages and *P. aeruginosa* PASS1. Dual RNA-sequencing of host-pathogen was undertaken to profile RNA expression simultaneously in the pathogen and the host during *P. aeruginosa* infection. Following establishment of infection in zebrafish embryos with PASS1, 3 days post infection (dpi), there were 6739 genes found to be significantly differentially expressed in zebrafish and 176 genes in PASS1. A range of virulence genes were upregulated in PASS1, including genes encoding pyoverdine biosynthesis, flagellin, non-hemolytic phospholipase C, proteases, superoxide dismutase and fimbrial subunits. Additionally, iron and phosphate acquisition genes were upregulated in PASS1 cells in the zebrafish. Transcriptional changes in the host immune response genes highlighted phagocytosis as a key response mechanism to PASS1 infection. Transcriptional regulators of neutrophil and macrophage phagocytosis were upregulated alongside transcriptional regulators governing response to tissue injury, infection, and inflammation. The zebrafish host showed significant downregulation of the ribosomal RNAs and other genes involved in translation, suggesting that protein translation in the host is affected by PASS1 infection.

## Introduction

Cystic fibrosis (CF) is an autosomal recessive disorder caused by mutations in the cystic fibrosis transmembrane conductance regulator gene (Freedman et al., 1999; Phennicie et al., 2010). It is most prevalent among the Caucasian population, affecting 1 in 2,500 new-borns (Freedman et al., 1999). CF lung disease is the major cause of morbidity and mortality among CF patients and is a result of colonization and infection of airways with bacteria, fungi, and viruses (Cantin et al., 2015). In early childhood, the CF lung is mainly colonized by *Staphylococcus aureus* and *Haemophilus influenzae*, although later in a CF patient's life, *P. aeruginosa* typically takes over as the dominant pathogen (Davies, 2002).

*P. aeruginosa* is a versatile Gram-negative microorganism found commonly in both terrestrial and aquatic environments (Whitehead et al., 2006; Sousa and Pereira, 2014). Its 6.3 Mb sized genome supports its metabolic versatility and, consequently, its adaptability to diverse environments (Blázquez et al., 2006). This opportunistic pathogen can cause acute and chronic infections in immunocompromised people, such as AIDS sufferers and neutropenic patients undergoing chemotherapy, and patients with injuries, catheters, burn wounds, and non-CF-associated pulmonary infections (Lyczak et al., 2000; Papaioannou et al., 2013). *P. aeruginosa* infection often becomes the major cause of morbidity and mortality in CF patients (Folkesson et al., 2012).

The respiratory pathogenesis of *P. aeruginosa* can be attributed to an array of key virulence factors, including flagella, type III secretion system, phenazines, the iron scavenging siderophores pyochelin and pyoverdine, lipopolysaccharide, elastase, alkaline proteases, hemolysins (phospholipase and lecithinase), cytotoxins (leukocidin), and exotoxin A (Sadikot et al., 2005; Gellatly and Hancock, 2013). *P. aeruginosa* chronic infections in the CF lung also provoke aggressive inflammatory reactions, such as the host neutrophilic response which releases oxidants and enzymes detrimental to the host tissue (Davies, 2002; Phennicie et al., 2010; Gellatly and Hancock, 2013).

The pathogenesis of *P. aeruginosa* has been studied using diverse model host organisms including *Dictyostelium discoideum, Arabidopsis thaliana, Caenorhabditis elegans, Drosophila melanogaster, Galleria mellonella*, rodents, and zebrafish (Clatworthy et al., 2009). Zebrafish has been previously used to study aspects of pathogenesis of *P. aeruginosa* (Phennicie et al., 2010; Diaz-Pascual et al., 2017), as well as other bacterial pathogens including *Salmonella typhimurium, S. aureus, Burkholderia cenocepacia, H. influenzae, Leptospira interrogans*, and *Listeria monocytogenes* (Meijer and Spaink, 2011).

Zebrafish is a teleost fish with a total genome size of 1.412 gigabases (van der Sar et al., 2003; Howe et al., 2013). Genome comparison has revealed that ~70% of human genes have a clear zebrafish ortholog (Howe et al., 2013). Remarkable similarities have been observed with human transcriptional regulators, immune effectors, immune recognition systems, defense signaling pathways, and macrophage lineages (Cui et al., 2011; Meijer and Spaink, 2011; Hall et al., 2013) which make zebrafish a good model for studying host-pathogen interaction.

There are several other advantages in using zebrafish embryos as a model organism for study of human infections. These include ease in handling, low cost, rapid development and the ability of a single pair of adult zebrafish to produce hundreds of offspring every week (Lessman, 2011; Meijer and Spaink, 2011). It is optically transparent and the availability of transgenic lines with fluorescently marked immune cells allows for real-time visualization of *in vivo* microbe-phagocyte interactions at high resolution throughout the organism (Clatworthy et al., 2009; Lessman, 2011; Meijer and Spaink, 2011).

In zebrafish, the innate and adaptive immune systems develop sequentially. The innate immune response is developed at the embryonic and early larval stages with early lymphocytes making their first appearance in the 4-day-old larvae, and a full adaptive immune system developing at about 3 weeks of age (Torraca et al., 2014). The innate immune system is the host's first line of defense against infections and includes physical barriers, cellular, and humoral components such as complement and acute phase proteins (Cui et al., 2011; van Soest et al., 2011). It is responsible for early recognition of pathogens and triggering an appropriate pro-inflammatory response (Mogensen, 2009; van der Vaart et al., 2012; Torraca et al., 2014). The main phagocytic cell types of the innate immune system are macrophages and neutrophils (Sieger et al., 2009; Torraca et al., 2014). In zebrafish embryos, as early as one day post fertilization, functional macrophages are capable of sensing and responding to microbial infections (Meijer and Spaink, 2011). Both neutrophils and macrophages can lead to bacterial clearance by engulfing and killing bacterial pathogens. The bacterial pathogens shown to be engulfed by these phagocytic cells include *P. aeruginosa* and *S. aureus* (Brannon et al., 2009; Clatworthy et al., 2009; Sieger et al., 2009).

Global gene expression studies of wild-type zebrafish embryos using a zebrafish microarray have been conducted following static immersion with *Edwardsiella tarda, Escherichia coli, P. aeruginosa* strain PA14 and strain PAO1, whereas systemic infection has been studied with *E. tarda* and *S. typhimurium* (Ordas et al., 2011; van Soest et al., 2011). Infection in zebrafish embryos is often established via injection into the blood circulation, and response to infection with various bacterial pathogens has been subjected to microarray analysis (Stockhammer et al., 2009; van der Sar et al., 2009; van Soest et al., 2011; van der Vaart et al., 2013; Lima et al., 2014). Specifically, zebrafish embryos are usually microinjected with bacterial pathogens directly into the blood circulation at 1-3 day post fertilization, mostly using the posterior blood island or into the duct of Cuvier, a wide blood circulation valley on the yolk sac connecting the heart to the trunk vasculature (Meijer and Spaink, 2011).

RNA sequencing (RNA-Seq) has previously been used to study innate immune response of zebrafish embryo to systemic *S. typhimurium* infection (Ordas et al., 2011). Ordas et al. (2011) used a combination of Tag-Seq, RNA-Seq and microarray transcriptome data to compile an annotated reference set of infection-responsive genes in the zebrafish embryos. These included genes encoding transcription factors, signal transduction proteins, cytokines and chemokines, complement factors, proteins involved in apoptosis and proteolysis, proteins with antimicrobial activities, as well as many known or novel proteins not previously linked to the immune response.

Despite the importance of *P. aeruginosa* pathogenesis in CF lung infection, the use of zebrafish as an alternative model to understand host-pathogen interaction until recently had remained unexplored. Diaz-Pascual et al. (2017) have recently profiled the global proteome of both zebrafish and *P. aeruginosa* PAO1 following establishment of *P. aeruginosa* PAO1 infection via immersion and injection (Diaz-Pascual et al., 2017). However, the interaction of zebrafish and *P. aeruginosa* remains to be elucidated at a global transcriptome scale. Hence, the aim of this study was to investigate the interaction of zebrafish embryos with the virulent *P. aeruginosa* CF isolate PASS1 (Penesyan et al., 2015) by visualizing macrophage-PASS1 interaction and analyzing the simultaneous global gene expression profiles of both organisms via RNA sequencing (RNA-Seq). To our knowledge, this is the first study describing the dual transcriptome of *P. aeruginosa*-zebrafish interaction.

## Methods

### *P. aeruginosa* strain and growth conditions

The bacterial strains used in this study were *P. aeruginosa* PASS1 (Penesyan et al., 2015) obtained from the sputum of a 40-year old female patient and yellow fluorescent protein (YFP)-tagged *P. aeruginosa* PASS1 (Kaur et al., 2015). The strains were maintained in a glycerol stock at −80°C, and prior to each experiment, were grown on Luria Bertani (LB) agar plates and incubated at 37°C till isolated colonies were obtained. The isolated colonies were then cultured in LB broth overnight at 37°C with constant shaking at 150 rpm. Cells from overnight cultures were washed, pelleted at 6,000 *g* for 10 min at 4°C and resuspended into sterile PBS. The cell concentration was estimated by measuring the optical density at 600 nm. Following estimation of cell concentration, the cells were diluted in PBS to an optical density OD_600_ of 2.0 which corresponds to 4.08 × 10^8^ CFU/mL. For visualization of the bacterial suspension during injection of zebrafish embryos an aliquot of phenol red sodium salt stock solution was added to a final concentration of 0.01%.

### Visualization of PASS1-YFP infection in zebrafish embryos by confocal microscopy

The injection apparatus for the zebrafish embryos was set up as described by Brudal et al. (2014). Zebrafish embryos derived from adults of the *Tg*(*mpeg1:Gal4, UAS;mCherry-CAAX*) (Ellett et al., 2011) were manually dechorionated and maintained at 29°C prior to injection at 48 hours post fertilization (hpf). The embryos were anesthetized with 0.005 w/vol % ethyl 3-aminobenzoate methanesulfonate (Tricaine) for 1–2 min and placed on 2% agarose plates for injection. PASS1-YFP cells (in a volume of 0.7 to 1 nl) were microinjected into the duct of Cuvier, as visually ascertained under the stereomicroscope. Infected embryos were returned to a petri dish with fresh embryo medium and incubated for 6 h at 29°C prior to confocal microscopy. A mock-infection with only sterile PBS was also set-up for comparison with the PASS1-YFP infection. Prior to confocal microscopy zebrafish embryos were anesthetized with Tricaine as described above, and then transferred to a glass bottom petri dish with glass cover slip containing a mixture of embryo water (Zebrafish embryo medium, 2011) and Tricaine. The anesthetized embryos in the petri dish were then covered with 1.3% low-melting-point agarose. Confocal microscopy was performed with an Olympus Fluoview FV1000 IX81 inverted confocal microscope.

### RNA extraction and RNA-seq transcriptomics

The zebrafish embryos derived from adults of the AB wild-type line were infected with PASS1 or mock-infected with PBS with microinjection into the duct of Cuvier (48 hpf) according to the procedure described above. Infected embryos were returned to a petri dish with fresh embryo medium and incubated for 3 dpi at 29°C prior to RNA isolation. At 3 dpi, 9 randomly chosen zebrafish embryos from each group were euthanized by a prolonged immersion in an overdose of 50 mg/L Tricaine solution and transferred into 1.5 ml Eppendorf tubes. Three embryos were then pooled to represent one sample to allow for sufficient amount of starting material for RNA isolation. The embryo water was replaced by RNAlater (Ambion) immediately after transfer of embryos to fresh 1.5 ml Eppendorf tubes. The samples were kept at 4°C until RNA was isolated. For the extraction of total RNA, RNAlater was replaced with 600 μl of Qiazol, and the tissue was homogenized using a pestle motor followed by drawing of sample with a needle 5 times till tissue was completely homogenized. As a control, RNA was isolated from PASS1 cultures grown in LB to an OD_600_ = 1.0. For both the zebrafish and bacterial culture, RNA extractions were performed using the miRNeasy Mini kit (Qiagen) according to the manufacturer's protocols. An additional DNase treatment was performed with a TURBO DNA-free kit (Ambion) according to the manufacturer's protocols. The concentration of the extracted RNA was measured using a NanoDrop Spectrophotometer. The total RNA samples were subjected to ribosomal RNA (rRNA) depletion, zebrafish samples mock-infected with PBS were treated with Ribo-Zero Gold rRNA Removal Kit (Human/Mouse/Rat) (Illumina), zebrafish infected with PASS1 were treated with Ribo-Zero rRNA Removal Kit (Gram-negative Bacteria) (Illumina) followed by Ribo-Zero Gold rRNA Removal Kit (Human/Mouse/Rat) and the PASS1 sample grown in LB was treated with Ribo-Zero rRNA Removal Kit (Gram-negative Bacteria). The depletion steps and subsequent 125 bp paired-end RNA Sequencing on a HiSeq2500 (Illumina) were performed at the Australian Genome Research Facility (Melbourne, Australia).

### Bioinformatic analyses of transcriptomic data

Sequencing data were assessed for quality using FastQC software (Babraham Bioinformatics). The transcriptomes of zebrafish infected with PASS1 and PBS were mapped against the zebrafish genome (Ensembl). Bacterial transcriptomic data were mapped against the PAO1 (NCBI) genome. Transcriptome mapping was undertaken with TopHat2 and normalized based on FPKM differential expression calculation using Cuffdiff (Trapnell et al., 2012).

Significantly differentially expressed genes in PASS1 (*p* ≤ 0.01 and log_2_ fold-changes cut-off −1≥ to ≤1) were mapped to annotated pathways and to cluster of orthologous groups of *P. aeruginosa* strain PAO1, obtained from the *Pseudomonas* Database (Winsor et al., 2016).

Significantly differentially expressed genes in zebrafish (*p* ≤ 0.01 and log_2_ fold-changes cut-off −1≥ to ≤1) were functionally annotated using Ingenuity Pathway Analysis (Ingenuity Systems Inc., Redwood City, CA). A total of 3,238 differentially expressed genes were successfully mapped (Table S6). Functional annotation and gene ontology (GO) classification was conducted separately of the upregulated and downregulated genes in zebrafish-*P. aeruginosa* PASS1 infection using DAVID (The Database for Annotation, Visualization, and Integration Discovery) version 6.8 (Huang da et al., 2009a,b).

## Results and discussion

### Confocal laser scanning microscopy of macrophage - *P. aeruginosa* PASS1 interaction in zebrafish

Macrophages are important effector cells of the innate immune response that can rapidly phagocytose bacteria and alert the immune system to danger (Kline et al., 2009). We have previously generated a (YFP)-labeled derivative of the CF isolate *P. aeruginosa* PASS1, PASS1-YFP (Kaur et al., 2015). The PASS1-YFP cells were injected into the Duct of Cuvier of transgenic zebrafish embryos *Tg(mpeg1:Gal4, UAS;mCherry-CAAX*) (Ellett et al., 2011) which produce mCherry-labeled macrophages. This enabled the analysis of macrophage behavior in zebrafish by confocal laser scanning microscopy (CLSM) visualization.

**Figure 1:**
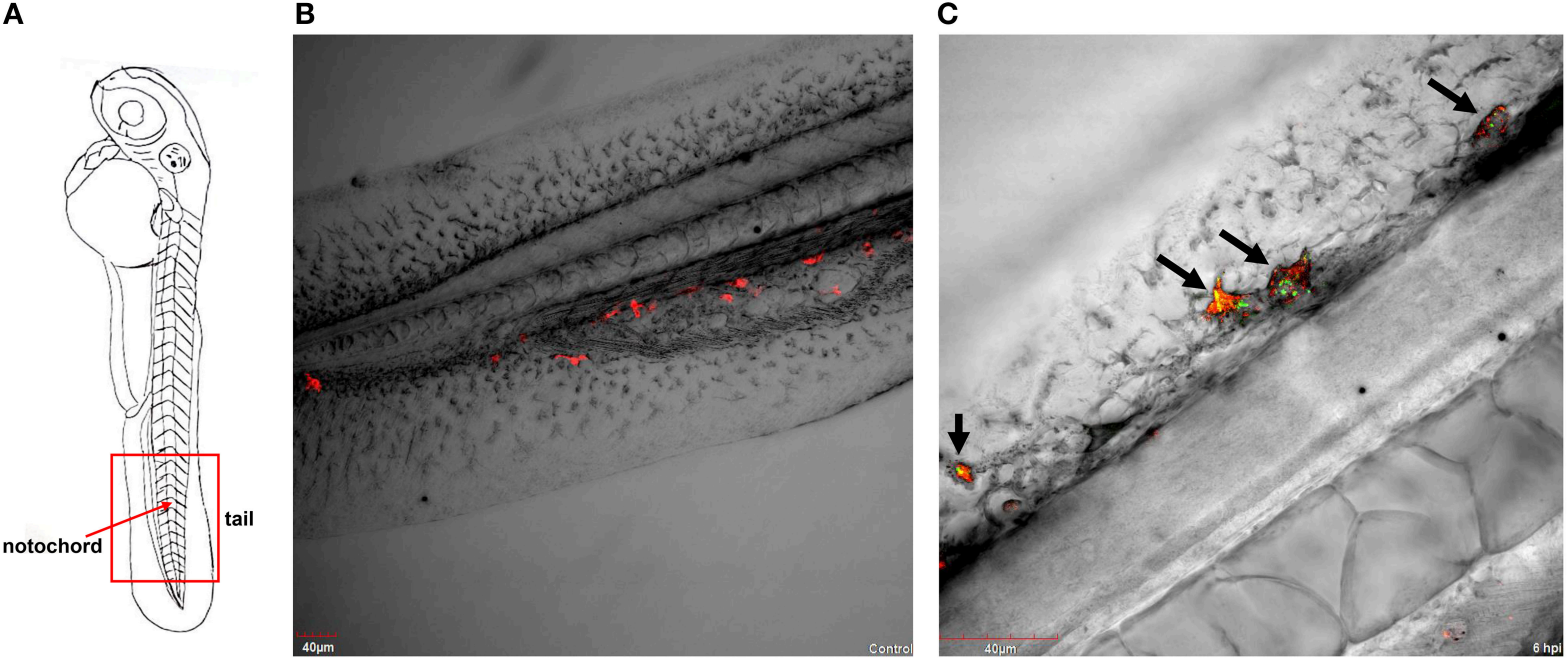
*P. aeruginosa* PASS1-macrophage interaction in zebrafish at 6 hpi. **(A)** A schematic view of a zebrafish embryo. The red box represents the tail region, and the red arrow indicates vertebrate notochord. Confocal laser scanning microscopy of transgenic embryos of *Danio rerio Tg*(*mpeg1:Gal4, UAS;mCherry-CAAX*) injected into the duct of Cuvier with **(B)** phosphate buffered saline and with **(C)** YFP-labelled *P. aeruginosa* PASS1 at 48 hpf. **(B)** Post-infection with phosphate buffered saline, macrophages are localized in the tail region of the vertebrate. **(C)** The black arrows on the vertebrate notochord in the tail region of zebrafish embryos indicate either an association or engulfment of *P. aeruginosa* PASS1-YFP cells (in green) by macrophages (in red).

Strikingly, within 6 h post infection (hpi), *P. aeruginosa* PASS1-YFP cells were predominantly found to be associated or engulfed by macrophages (Figures 1A–C). Previous studies have shown that macrophages can kill both Gram-positive and Gram-negative bacteria, including *P. aeruginosa*, via phagocytosis (Brannon et al., 2009). Brannon et al. (2009) have shown the phagocytosis of *P. aeruginosa* strains PAO1 and PAK by macrophages to occur within 2 hpi (Brannon et al., 2009).

A central advantage of the zebrafish embryo model is the ability to monitor infection at a detailed cellular level in real time. Our CLSM results corroborate previous observations that macrophages are capable of phagocytosing and killing *P. aeruginosa* (Tang et al., 1995; Brannon et al., 2009). Earlier studies have used the laboratory strains PAO1 and PAK, this is the first zebrafish study to use a CF isolate. Our previous work showed that PASS1 is non-mucoid and has a mutation in the *lasR* gene, and displays significant phenotypic differences compared with PAO1, including increased biofilm formation and production of virulence factors such as phenazines (Penesyan et al., 2015).

### Generation of a dual host-pathogen transcriptome

**Figure 2:**
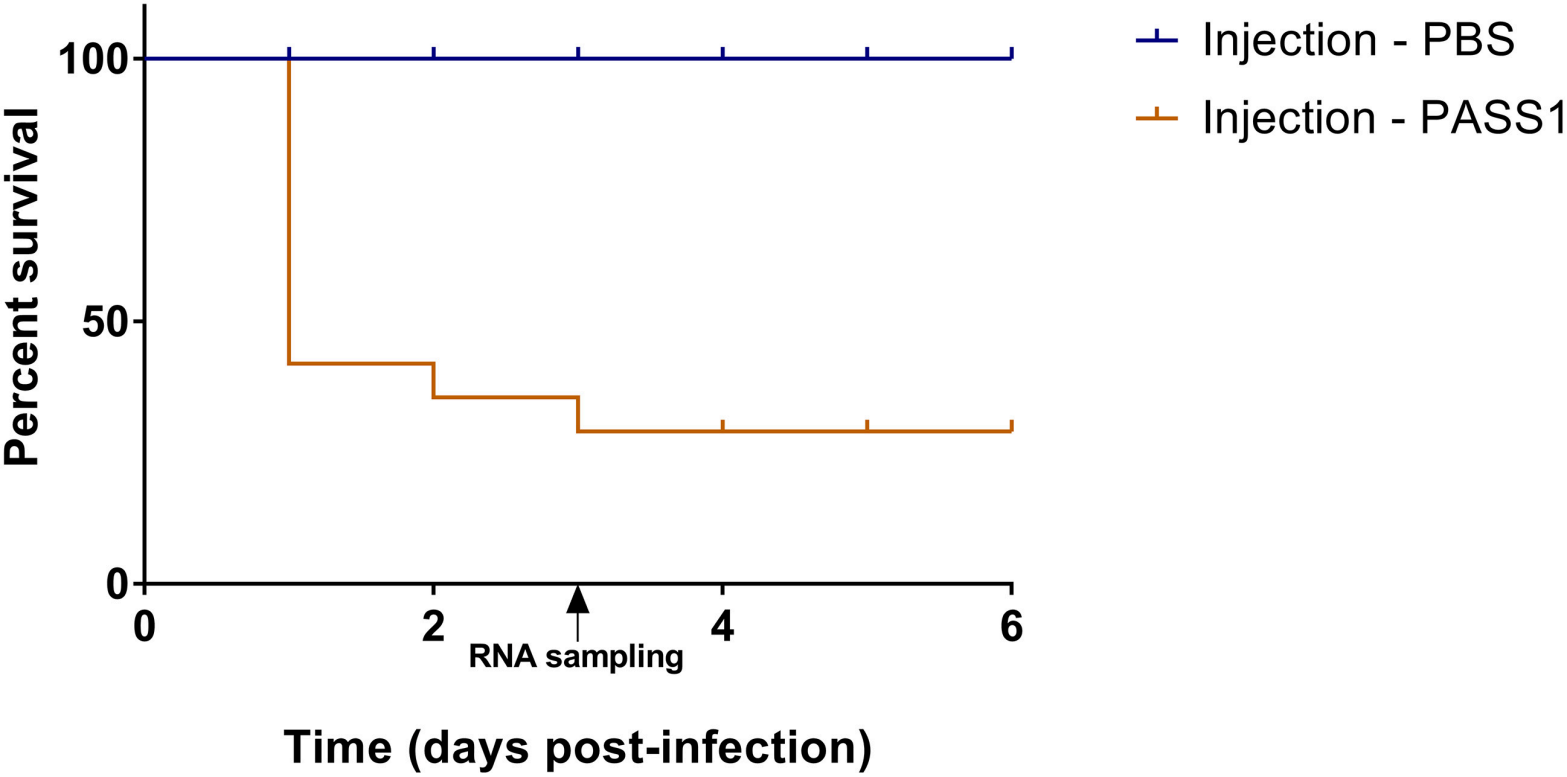
Percentage survival of zebrafish embryos infected with *P. aeruginosa* PASS1. Kaplan-Meier representation of the survival of zebrafish embryos infected with PASS1 and mock-infected with PBS via injection into the duct of Cuvier.

The use of a zebrafish embryo model allows the possibility of global gene expression analysis of both host and microbe in parallel. This provides an opportunity to investigate the molecular mechanisms of the interaction between the host innate immune system and the pathogen. The survival of zebrafish embryo infected with PASS1 displayed increased mortality within 24 h of infection followed by gradual decrease in embryo survival rate till 3 days post infection (dpi) (Figure 2). To investigate host-pathogen interaction prior to increase in mortality, we isolated total RNA from PASS1-infected zebrafish (3 dpi), and RNA-Seq was used to examine the zebrafish and PASS1 transcriptomes in parallel. The infected zebrafish transcriptome was compared with phosphate buffered saline (PBS)-injected zebrafish as a negative control. The transcriptome of *P. aeruginosa* PASS1 in zebrafish was compared with PASS1 grown in Luria-Bertani (LB) culture medium.

A total of 214,749,236 sequence reads were generated from the total RNA extracted from three independent biological replicates of PASS1-infected zebrafish. Around 53.2% of the reads aligned with the *P. aeruginosa* reference genome while 90% aligned with the zebrafish reference genome (Table 1).

**Table 1:** Summary of *P. aeruginosa* PASS1 and zebrafish embryos mapped reads at 3 days post-infection.

**Condition (3 dpi)**	**Replicate**	**Mapped reads**	**Percentage aligned (%)**
**SEQUENCE READS MAPPED TO ZEBRAFISH**			
PASS1-infected zebrafish	1	39709254	90.2
	2	43085353	89.8
	3	40410278	90.0
PBS-infected zebrafish	1	38104344	90.2
	2	40702248	90.3
	3	39929890	90.0
**SEQUENCE READS MAPPED TO** ***P. aeruginosa*** **PAO1**			
PASS1-infected zebrafish	1	27297	52.5
	2	15089	53.1
	3	21174	54.0
PASS1 grown in Luria-Bertani medium	1	12449136	94.0
	2	16617581	94.3
	3	15580588	94.6

### Whole-cell transcriptome analysis of *P. aeruginosa* infected into zebrafish

**Figure 3:**
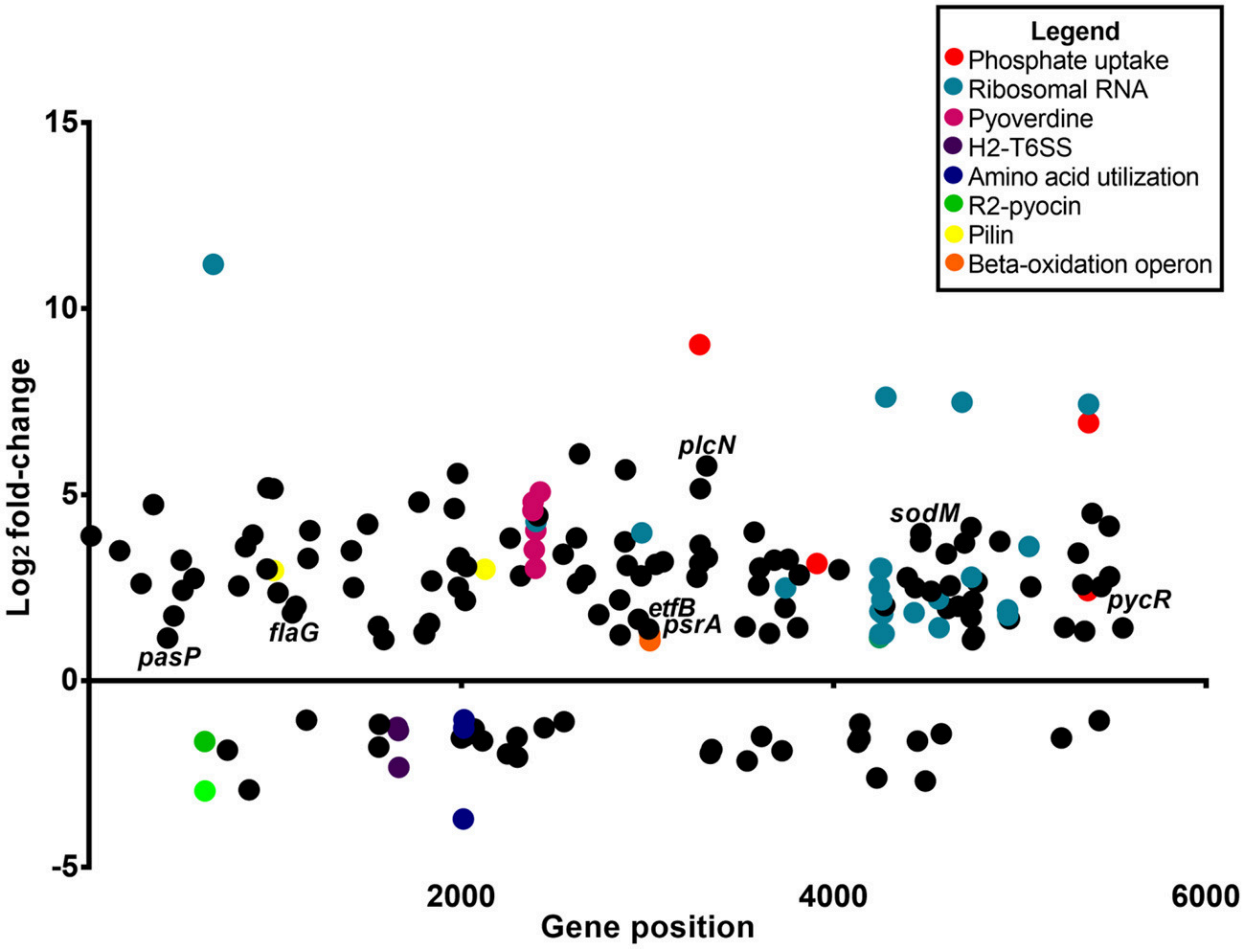
Differential gene expression of *P. aeruginosa* PASS1 in zebrafish compared to PASS1 cells grown in Luria-Bertani medium. Each dot represents a gene within the *P. aeruginosa* PASS1 genome (x-axis) and its fold-change (log_2_) expression *in vivo*, 3 dpi. Only significantly differentially expressed genes are shown (*p* ≤ 0.01 and log_2_ fold-change cut-off −1≥ to ≤1).

Analysis of the *P. aeruginosa* transcriptome data revealed 176 genes to be differentially expressed in *P. aeruginosa* within the infected zebrafish (*p* ≤ 0.01 and log_2_ fold-changes cut-off −1≥ to ≤1) with 140 genes upregulated and 36 genes downregulated (Figure 3). Ribosomal RNA genes were the most upregulated transcripts in *P. aeruginosa* PASS1 during zebrafish infection (Figure 3). A number of studies have shown a correlation between growth rate and rRNA concentration (Bartlett and Gourse, 1994; Rang et al., 1999; Ramos et al., 2000; Schneider et al., 2003; Dennis et al., 2004; Benítez-Páez et al., 2012; Blazewicz et al., 2013). Additionally, translation initiation and elongation factor genes were also more highly expressed by PASS1 within the zebrafish (Table S1). This suggests that within the host there is a higher rate of protein synthesis and cell proliferation at 3 dpi compared to the late log (OD_600_ = 1.0) culture of PASS1 in LB medium.

### Expression of virulence genes in PASS1 cells in a zebrafish model

**Figure 4:**
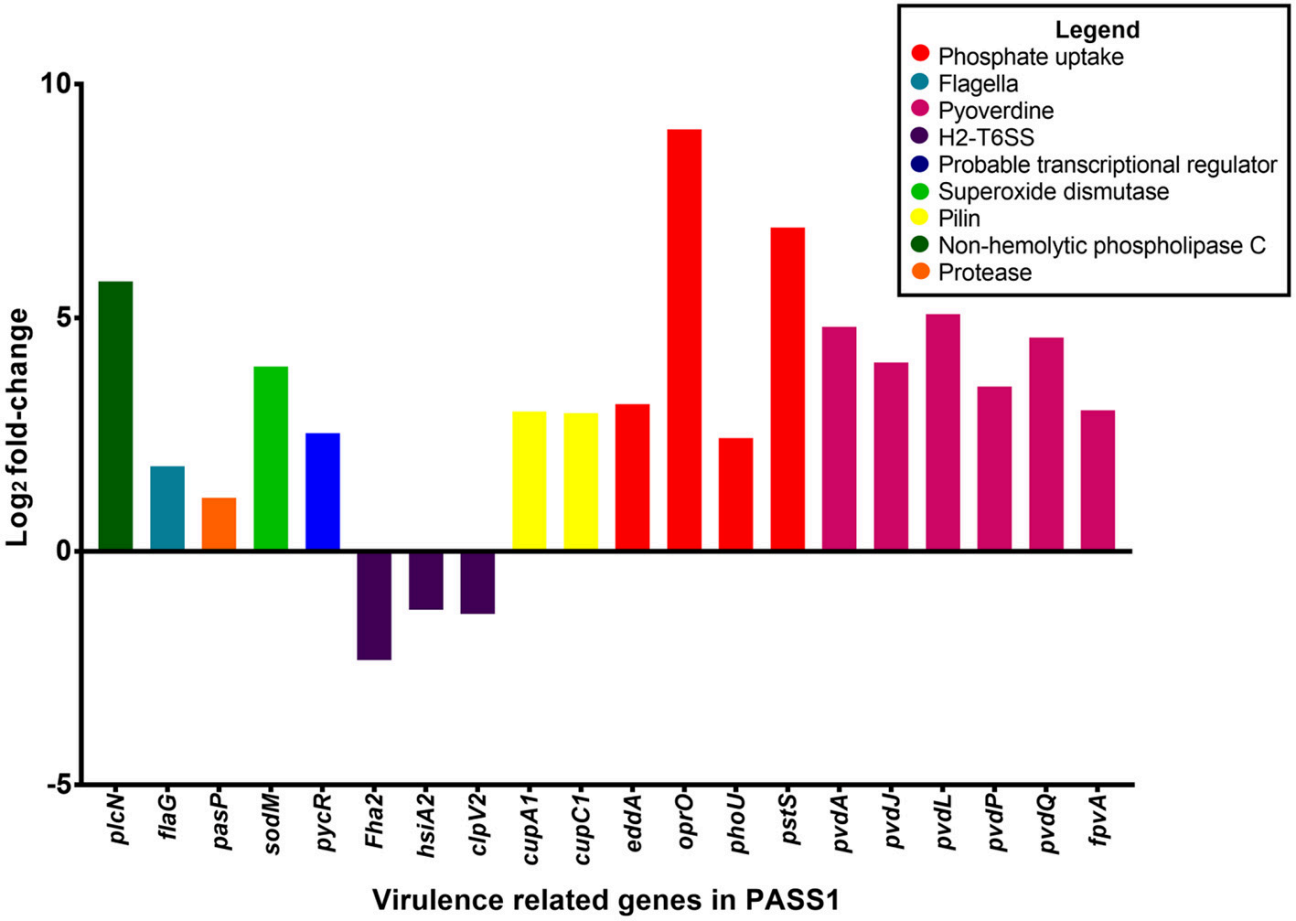
Differential gene expression of virulence-related genes of *P. aeruginosa* PASS1 during infection of zebrafish compared to PASS1 cells grown in Luria-Bertani medium. Log_2_ fold-change differential expression of known virulence genes (*p* ≤ 0.01 and log_2_ fold-changes cut-off −1≥ to ≤1).

Vertebrates are known to deplete both inorganic phosphate and iron in response to bacterial infection (Skaar, 2010). Consistent with this the *P. aeruginosa* phosphate transport and iron acquisition genes were highly expressed in zebrafish compared with PASS1 culture in LB (Figure 4). The pyrophosphate porin gene *oprO* and the phosphate-binding protein gene *pstS* were highly upregulated (9 log_2_ fold-change and 6.9 log_2_ fold-change, respectively). Phosphate regulation (*phoU*), and DNA degradation (*eddA*) genes were also upregulated. All four genes have been identified to be upregulated under phosphate limitation *in vitro* (Hancock and Brinkman, 2002; Bains et al., 2012). *P. aeruginosa* is able to obtain phosphate from the host cell membrane via hydrolysis of the phospholipids using phospholipases (Sadikot et al., 2005). The non-hemolytic phospholipase C gene was highly upregulated 5.8 log_2_ fold-change in *P. aeruginosa* PASS1 during zebrafish infection (Figure 4).

Also upregulated were iron scavenging systems of PASS1. Pyoverdine biosynthesis genes and the ferri-pyoverdine receptor gene of the PASS1 strain were significantly upregulated in zebrafish. *P. aeruginosa* synthesizes and secretes the siderophore pyoverdine to scavenge ferric iron from host to overcome iron limitation during infection (Lamont et al., 2002; Konings et al., 2013; Nguyen et al., 2014).

In addition, a range of known *P. aeruginosa* virulence genes were differentially expressed in the zebrafish host compared with the culture in LB medium. These include genes encoding flagella biogenesis and the PasP protease (Figure 4). PasP is an extracellular protease (Pelzer et al., 2015) able to cleave collagen, contributing to the loss of epithelial cells (Tang et al., 2009, 2013).

The *P. aeruginosa flaG* flagellin gene was upregulated 1 log_2_ fold-change in the zebrafish host. The delivery of bacterial flagellin into the mice macrophage cytosol has been shown to trigger the NLRC4 inflammasome which mediates activation of the protease, caspase-1 (upregulated in our data set by 1 log_2_ fold-change (Mariathasan et al., 2004; Sutterwala et al., 2007). The activation of caspase-1 promotes the secretion of the proinflammatory cytokines IL-1β and IL-18 as well as pyroptosis, a form of cell death induced by bacterial pathogens (Franchi et al., 2007).

Expression of the *cupA1* and *cupC1* genes, encoding fimbrial subunits of chaperone-usher type fimbriae, were both increased in PASS1 in zebrafish. These class of fimbriae are important tissue-specific adhesins in many pathogens, and are presumably playing a role in adhesion to zebrafish cells.

The *pycR* gene encoding a LysR-type transcriptional regulator was upregulated by 2.5 log_2_ fold-change. This regulator modulates expression of virulence factors such as lipase/esterase and biofilm formation, as well as genes implicated in lipid metabolism and anaerobic respiration (Kukavica-Ibrulj and Levesque, 2008) (Figure S1).

The *P. aeruginosa sodM* superoxide dismutase gene was upregulated by 4 log_2_ fold-change in zebrafish. SodM protects *P. aeruginosa* against toxic effects of superoxides (Iiyama et al., 2007), so the increased expression level of *sodM* may be a defensive response against oxidative killing in the zebrafish macrophage phagolysosome. Iron limitation has been reported to lead to an increase in SodM activity in *P. aeruginosa* (Chang et al., 2005), which may be an alternate explanation for increased *sodM* expression. All of the *P. aeruginosa* type VI secretion system genes (orthologous to PA1656-PA1671 of PAO1) encoded within the Hcp secretion island-2 (H2-T6SS) showed lower expression levels in zebrafish compared with the culture in LB medium, although not all of them were below the significance threshold (*p* ≤ 0.01) (Table S1). This type VI secretion system in PAO1 has been shown to be important in virulence in a worm model and in mammalian cell cultures, and its expression has been shown to be induced by the Fur regulator during iron limitation and by quorum sensing (Sana et al., 2012). However, our transcriptomic data indicates that PASS1 is responding to iron limited conditions, thus the downregulation of the H2-T6SS genes in our infected zebrafish model suggests that there are other unknown regulatory pathways governing expression of this secretion system.

### Other differentially expressed PASS1 genes in a zebrafish model

The PASS1 genes *faoA* and *foaB* located in the *fadBA5* β-oxidation operon were upregulated in the infected zebrafish. β-oxidative enzymes have been shown to be induced *in vivo* during lung infection in CF patients (Kang et al., 2008). *In vitro* studies have demonstrated that the *fadBA5* operon is required for phosphatidylcholine (PC) and fatty acid (FA) degradation (Kang et al., 2008; Turner et al., 2014). The lung surfactant consists of ~10% surfactant proteins and ~90% lipids with phosphatidylcholine (PC) accounting for ~80% of the lipids (Griese, 1999; Son et al., 2007). The most abundant lipids in the zebrafish embryo are cholesterol, PC, and triglyceride (Fraher et al., 2016). These lipids are processed within the yolk prior to mobilization to the embryonic body (Hölttä-Vuori et al., 2010; Fraher et al., 2016). The PASS1 *psrA* gene encoding a TetR family transcriptional regulator required for regulation of the *fadBA5* operon also showed increased expression in the zebrafish host (Figure S1). PsrA has been reported to also regulate the electron transfer flavoprotein B-subunit, *etfB* gene during stationary phase of bacterial growth (Kojic et al., 2005), and *etfB* also showed increased expression in PASS1 in zebrafish.

A variety of amino acid utilization genes, for example, *liuA, liuB*, and *liuE* encoding enzymes in the branched chain amino acid degradation pathway, showed decreased levels of expression in PASS1 cells in zebrafish. Conversely, a variety of PASS1 amino acid biosynthesis genes showed increased levels of expression in the zebrafish infection model. This most likely reflects the availability of amino acids in LB medium that was used for the *in vitro* control PASS1 culture.

The PA0622 and PA0623 genes in the bacteriocin R2 pyocin gene locus (Waite and Curtis, 2009; Purschke et al., 2012) were downregulated in PASS1 cells in the zebrafish model (Figure 3, Figure S2). This gene cluster has been implicated in the production of a cryptic prophage endolysin that mediates *P. aeruginosa* explosive cell lysis (Turnbull et al., 2016). This cell lysis results in the production of extracellular DNA that facilitates biofilm formation. Other biofilm-related genes, such as GacS/GacA and RetS/LadS two-component systems and quorum-sensing systems including *las, rhl*, and *pqs* (Rasamiravaka et al., 2015) were not significantly differentially expressed (Table S1).

### Whole-cell transcriptome analysis of zebrafish embryos infected with *P. aeruginosa* PASS1

**Figure 5:**
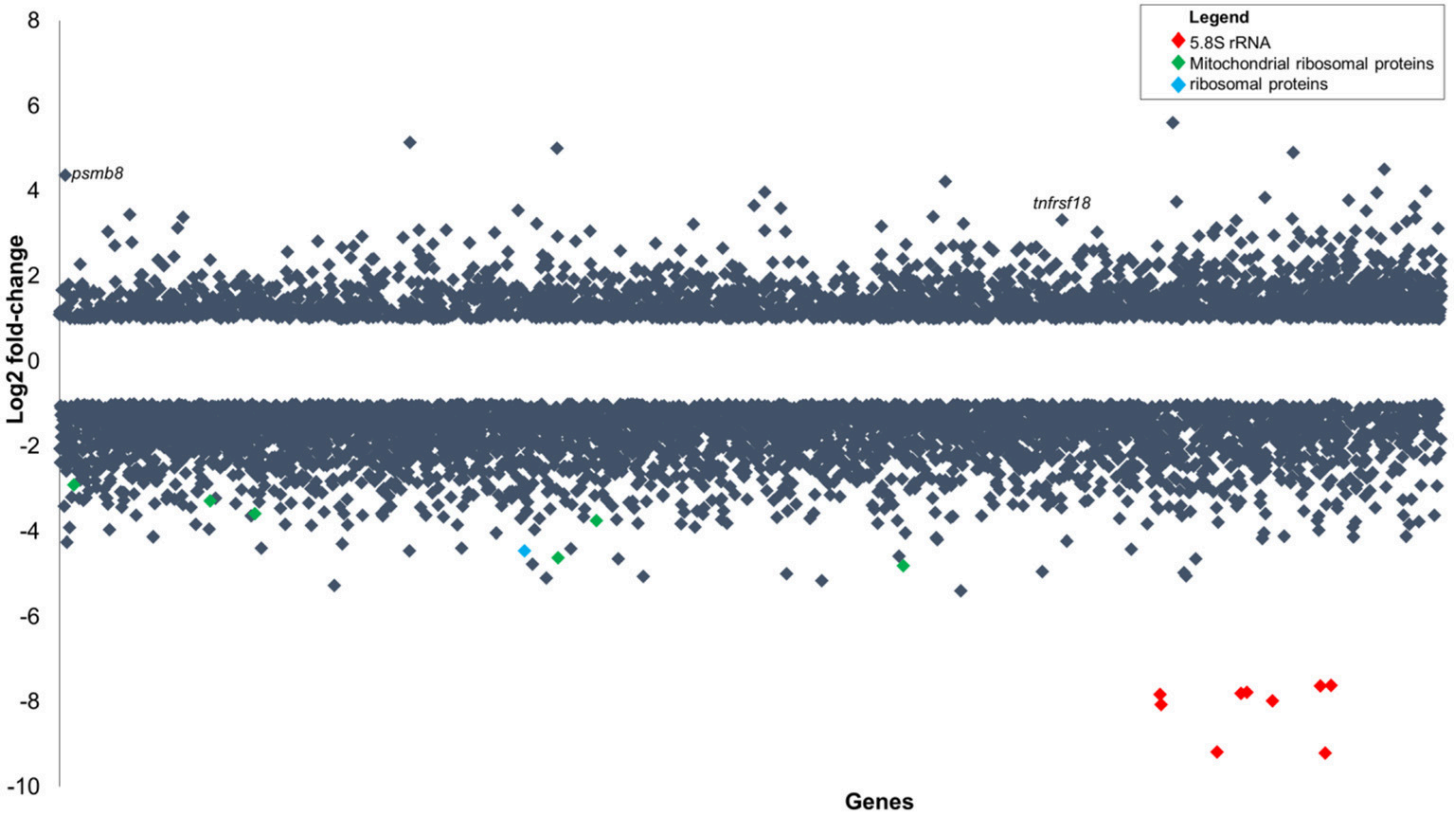
Gene expression changes in zebrafish embryos (log_2_ fold-change) infected with *P. aeruginosa* PASS1 compared to zebrafish embryos injected with phosphate buffered saline. Each dot represents a gene within the zebrafish genome (x-axis) and its fold-change (log_2_) expression 3 dpi (*p* ≤ 0.01 and log_2_ fold-changes cut-off −1≥ to ≤1).

The transcriptome of zebrafish infected with PASS1 was compared with PBS-injected zebrafish to identify genes upregulated in response to *P. aeruginosa* infection. RNA-Seq analysis revealed 6,739 genes to be differentially expressed (*p* ≤ 0.01 and log_2_ fold-changes cut-off −1≥ to ≤1). This represents a quarter of the protein-encoding genes in the zebrafish genome, suggesting that there is a dramatic transcriptional response to infection. Of the differentially expressed genes, 2,510 were found to be upregulated and 4,229 were downregulated. The complete list of genes is provided in Table S2, and their log_2_ fold change in expression (*p* ≤ 0.01, log_2_ fold-change cut-off −1≥ to ≤1) are shown graphically in Figure 5.

Two of the most highly upregulated genes in the infected zebrafish are *PSMB8* (4.4 log_2_ fold-change) and *TNFRSF18* (3.3 log_2_ fold-change) (Figure 5). The *PSMB8* gene has been linked to a number of auto-inflammatory diseases and found to be induced during the innate immune response of zebrafish to bacterial infection (Meijer and Spaink, 2011; Warnatsch et al., 2013). The *TNFRSF18* gene is part of the TNF receptor signaling family and plays a role in anti-apoptotic signaling via TRAF2 (upregulated 0.4 log_2_ fold-change), which is thought to be involved in protection of lymphocytes against activation-induced cell death (Donaldson et al., 2005). Mycobacterial infection of zebrafish has suggested that TNF receptor signaling mediates resistance against mycobacteria (Meijer and Spaink, 2011). *PSMB8* and *TRAF2* genes may play a similar role in the zebrafish immune response against *P. aeruginosa* PASS1.

The most significantly downregulated genes in the zebrafish infected with *P. aeruginosa* PASS1 were the 5.8S rRNA genes (Figure 5). RNA isolated from both the infected and uninfected control cells underwent an rRNA depletion step, which makes it difficult to conclusively draw inferences about translation, as the differences in the 5S rRNA abundance could be due to artifacts introduced by the depletion process. Nevertheless, 22 mitochondrial ribosomal proteins, 9 ribosomal proteins, as well as several ribosomal proteins modifying enzymes all showed decreased expression in the infected zebrafish cells compared with the uninfected control (Table S2). This suggests that translation in the zebrafish is negatively impacted by the bacterial infection.

The downregulation of transcription of rRNA genes and ribosomal protein genes has been previously reported to occur due to intracellular and extracellular stressors (Xiao and Grove, 2009; Hayashi et al., 2014). Stress leads to the induction of processes such as cell cycle arrest, apoptosis or autophagy (Naora, 1999; Gupta et al., 2012; Hayashi et al., 2014). Consistent with this the PASS1-infected zebrafish transcriptome was significantly enriched in transcripts related to organismal injury and cell death (Figure S3).

PASS1-infected zebrafish displayed decreased expression of the prohibitin 2 (PHB2) mitochondrial protein, a coordinator/communication protein for cell division, metabolism, and cell death (Bavelloni et al., 2015). Proteins known to interact with PHB2, including transcription factors ATF2, MEF2A, TEAD3, DNA modifying proteins, SIRT2, HDAC5, RNF2, protease, AFG3L2, RNA binding/ processing proteins, AGO3, DDX20, cell cycle, KIF23, cytoskeleton/structural protein, NUP93, signal transduction, ADRB2, ATP5B, COX4I1, cellular respiration protein, COX6C, mitochondrial transport/translation TIMM50 were also significantly downregulated in PASS1-infected zebrafish, suggesting that PASS1 infection is impacting a swathe of activities linked to PHB2.

### Cellular and humoral innate immune response in zebrafish infected with PASS1

Pathway analysis of the zebrafish transcriptome using the Ingenuity package revealed that several canonical pathways within the category of cellular and humoral innate immunity were highly enriched in zebrafish upon infection (Figure S4). These included phagosome maturation, leukocyte extravasation signaling, Fcɤ receptor-mediated phagocytosis in macrophages and monocytes, CXCR4 signaling, clathrin-mediated endocytosis signaling, IL-8 signaling, caveolar-mediated endocytosis signaling, fMLP signaling in neutrophils, production of nitric oxide and reactive oxygen species in macrophages, and macropinocytosis signaling.

**Figure 6:**
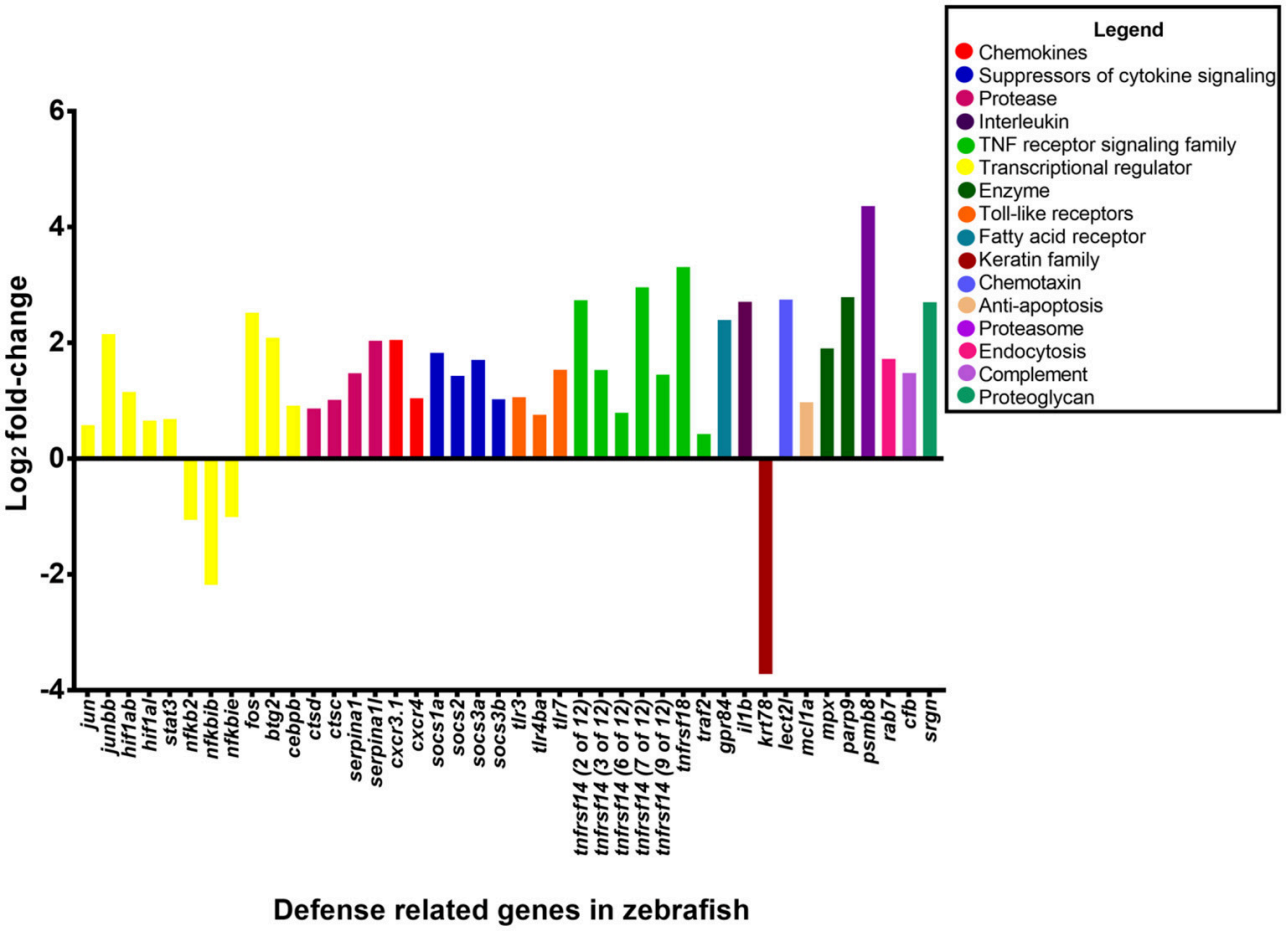
Expression of host defense-related genes in response to infection with *P. aeruginosa* PASS1. The upregulated genes in zebrafish infected with PASS1 (*p* ≤ 0.01 and log_2_ fold-change cut-off −1≥ to ≤1).

In the leukocyte extravasation signaling pathway, the chemokines *CXCR3* and *CXCR4* was upregulated by 2 log_2_ fold-change and 1.1 log_2_ fold-change, respectively (Figure 6). The leukocyte extravasation signaling pathway involves the movement of leukocytes out of the circulatory system and toward the site of tissue damage and infection. Members of the CXC chemokine family have a role in inducing neutrophil recruitment (Gellatly and Hancock, 2013). Previously, the CXC chemokine family has been shown to be involved in the inflammatory response in mice to *Pseudomonas* lung infection (Tsai et al., 2000). These chemokines are presumably involved in enhancing migration of leukocytes to the site of bacterial infection.

Toll like receptors (TLRs) which are expressed by neutrophils and macrophages play a key role in recognition of bacterial ligands (Lloyd et al., 2007; Mittal et al., 2014). *TLR3, 4*, and *7* were significantly upregulated in the zebrafish transcriptome following PASS1 infection (Table S2). In particular, TLR4, which can recognize bacterial lipolysaccharide, plays a significant role in the response to *P. aeruginosa* infections in mammalian lungs (Campodónico et al., 2008). TLRs also play important roles in regulating phagocytosis at multiple steps including internalization and enhancement of phagosome maturation (Blander and Medzhitov, 2004; Kagan and Iwasaki, 2012). *RAB7* which is a mediator of late phagosome process (Flannagan et al., 2009) was upregulated by 1.7 log_2_ fold-change, suggestive of phagosomal activity against PASS1 inside the zebrafish host, which is consistent with interaction between PASS1 and phagosomes observed in our confocal microscopy (Figure 1).

Key transcriptional regulators in the acute phase response to tissue injury, infection, and inflammation, including *FOS, JUN*, and *STAT3* were upregulated, while the *NFKB2, NFKBIB, NFKBIE* regulators were downregulated in response to PASS1 infection (Moshage, 1997). Previous studies using zebrafish embryos have shown upregulation of *FOS* and *STAT3* in response to *S. typhimurium* and *M. marinum* (van der Vaart et al., 2012). The *JUN* and *FOS* transcriptional regulators together are known as activating protein 1 (AP-1), both are conserved between mammals and zebrafish (Meijer and Spaink, 2011; Ordas et al., 2011). AP-1 is involved in cellular expression, cell proliferation and differentiation, with its activation dependent on a variety of stress-related stimuli (Kim et al., 2003).

The intracellular suppressors of cytokine signaling (SOCS) genes *SOCS1, SOCS2*, and *SOCS3* were upregulated in the acute phase signaling pathway by 1.8-, 1.4-, and 1 log_2_ fold-change, respectively, in response to PASS1 infection. The SOCS proteins are important regulators of acute phase response and cytokine signaling pathways as they regulate the balance between pro- and anti-inflammatory signals during infection (Wang et al., 2011; Brudal et al., 2014).

### Identification of genes previously linked to cell infection

Based on the Ingenuity Pathway Analysis there were 233 differentially expressed genes related to infection of cells (Figure S5). The 233 differentially expressed genes comprised genes encoding enzymes, G-protein coupled receptors, ion channels, growth factors, kinases, ligand–dependent nuclear receptors, peptidases, transcription regulators, translational regulators, transmembrane receptors, and transporters. The genes with the highest expression changes (> 2 log_2_ fold-change) were the transmembrane receptor (tumor necrosis factor receptor superfamily 14, *TNFRS14*), poly (ADP-ribose) polymerase *PARP9*, transcription regulator (*BTG2*) and the serine protease inhibitor *SERPINA1* genes (Figure 6). This suggests a complex cascade of cellular events in response to *P. aeruginosa* infection.

### Other genes differentially expressed in zebrafish in response to bacteria

Functional analysis of upregulated genes using DAVID (Huang da et al., 2009a,b) (Tables S3, S4) showed enrichment within the GO category “response to bacterium.” The genes upregulated by 2 log_2_ fold-change included G protein-coupled receptor 84 (*GPR84*), leukocyte cell-derived chemotaxin 2 like (*LECT2L*), and the tumor necrosis factor receptors *TNFRSF18* and *TNFRSF14*. *GPR84* is expressed in leukocytes, monocytes and macrophages, and is known to play a critical role in immune regulation (Cha et al., 2013; Figure 6). The acute phase response LECT2 protein attracts neutrophils (Škugor et al., 2009) and studies of mammalian LECT2 indicate that it plays a role in immune regulation (Chen et al., 2010). Infection studies with *Aeromonas salmonicida* and *S. aureus* have shown high induction of *LECT2* in adult zebrafish (Lin et al., 2007). *MPX*, which is a zebrafish ortholog of the mammalian *MPO* gene was upregulated suggesting that there is presence of neutrophils at the site of infection which are undergoing apoptosis (Mathias et al., 2009).

### Comparison of zebrafish infection with various pathogens

Previously Ordas et al. (2011) have compared *Salmonella* infection of zebrafish embryos with *M. marinum* infection of adult zebrafish (Hegedus et al., 2009). Transcriptomic datasets from zebrafish were compared to identify the overlap between the up- and downregulated transcripts of the *Salmonella*- and *Mycobacterium*-infected zebrafish. This revealed 288 and 3 commonly up- or downregulated transcripts. Comparison of our dataset to both these infection studies revealed 47 and 1 common up- or downregulated transcripts (Table S5). The one downregulated gene common to these two datasets, as well as our PASS1 zebrafish embryo infection, was *KRT78*, involved in translation.

The common set of 47 upregulated genes included 19 previously implicated in the vertebrate immune response. The *MCL1A* gene was upregulated by 1 log_2_ fold-change protects against apoptosis during initial steps of differentiation in human macrophages (Arslan et al., 2012). Complement factor B in macrophages was upregulated by 1.5 log_2_ fold-change, and its expression was proposed to be facilitated by TLR3, TLR4, and TRIF (Li et al., 2011). The transcriptional regulator *CEBP*β was upregulated by 0.9 log_2_ fold-change, and it has been suggested to influence expression of the *IL-1*β gene (Didon et al., 2011), which, in turn, was upregulated by 2.7 log_2_ fold-change in our study, and is known to activate neutrophils and macrophages in bacterial phagocytosis (Didon et al., 2011). *HIF-1*α, a global regulator of macrophage and neutrophil inflammatory and innate immune functions that is stimulated by TLR4 (Zinkernagel et al., 2007), was upregulated by 0.7 log_2_ fold-change.

The gene encoding SRGN which interacts with inflammatory mediators such as IL-1β and TNF (Korpetinou et al., 2014) was upregulated by 1.4 log_2_ fold-change. The protease cathepsin C gene was upregulated by 1 log_2_ fold-change and is involved in the activation of granule serine peptidases in inflammatory cells (Turk et al., 2001). The cathepsin D protease gene was also upregulated (0.8 log_2_ fold-change) and has been implicated in macrophage apoptosis (Bewley et al., 2011).

Comparison of transcriptomic data from infection studies with different bacterial pathogens can thus be used to collectively define a common set of innate host genes expressed in response to infection. Comparison of zebrafish embryo infection studies with adult zebrafish infection studies provides an opportunity to dissect the innate immune response separate from the adaptive immune response. The generation of transcriptomics data investigating response to infection to various pathogens is valuable for future host-pathogen interaction studies as well as developing targeted therapeutics.

## Conclusions

**Figure 7:**
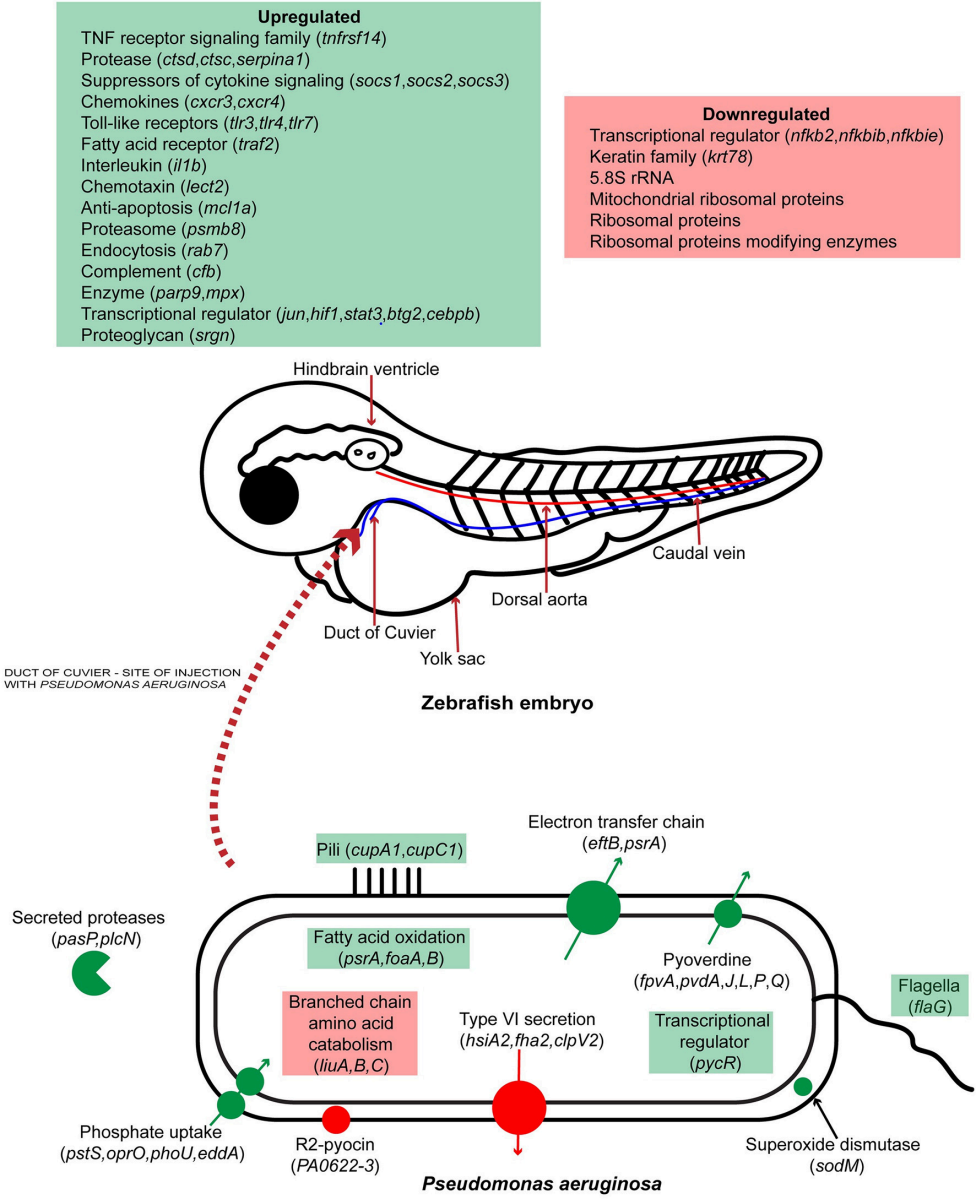
Schematic representation of host-pathogen interactions of zebrafish embryos infected with *P. aeruginosa* PASS1. Up and down regulated processes and genes are highlighted in green and red, respectively.

Previously, zebrafish was used as a model organism for *P. aeruginosa* infection by looking at the expression of specific immune related genes and *in vivo* interaction of the pathogen with phagocytes. In this study we report, for the first time, the simultaneous global gene expression of a zebrafish-*P. aeruginosa* systemic infection. RNA-Seq analysis has yielded a detailed view of both host and pathogen transcriptional responses. During infection, PASS1 displayed increased expression of an array of genes shown previously to be important in pathogenesis. We have also shown that phosphate and iron acquisition genes are significantly upregulated in PASS1, suggesting these are limiting nutrients within the zebrafish host. The response of zebrafish to PASS1 infection involved both humoral and cellular components of the innate immune system. Significant upregulation was observed for genes involved in bacterial recognition and clearance, inflammation and tissue injury. Based on the transcriptomic data, we present a schematic overview of the key response mechanisms in both host and pathogen during PASS1 infection of zebrafish embryos (Figure 7).

## Data availability statement

We provide two supplementary tables with the gene expression profile of *Pseudomonas aeruginosa* PASS1 and *Danio rerio* following host-pathogen interaction. The *P. aeruginosa* PASS1 and *Danio rerio* transcriptomic data can be found in Dryad under the accession number doi:10.5061/dryad.3vk38 at http://datadryad.org/reviewdoi=doi:10.5061/dryad.3vk38.

## Impact statement

This study represents the first global expression view of the molecular interactions between *Pseudomonas aeruginosa* and zebrafish. *P. aeruginosa* is an opportunistic pathogen that is the major cause of mortality among cystic fibrosis patients. Our transcriptomic data suggests that key virulence mechanisms for *P. aeruginosa* PASS1 in zebrafish include adherence to host cells via Cup fimbriae, iron and phosphate scavenging, protease cleavage of host proteins such as collagen, and superoxide dismutase as a defense mechanism against oxidative killing. The host gene expression during *P. aeruginosa* PASS1 infection shows upregulation of an array of genes including transcriptional regulators, toll-like receptors and chemokines to be involved in the initiation, and process of phagocytosis. Decreased expression levels of ribosomal RNAs and translation proteins suggests that protein translation in the host is impacted by bacterial infection.

## Ethics statement

All zebrafish experiments were performed with the approval of University of Oslo (Animal ethic approval number: 6981) and Macquarie University (Animal ethic approval number: 5201500513) animal ethics committee and Macquarie University Internal Biosafety Committee (NLRD 5201500584). Zebrafish embryos were utilized 48 hpf for all infection experiments.

## Author contributions

All authors listed have made a substantial, direct and intellectual contribution to the work, and approved it for publication.

### Conflict of interest statement

The authors declare that the research was conducted in the absence of any commercial or financial relationships that could be construed as a potential conflict of interest.

## Abbreviations

CF: cystic fibrosis
CLSM: confocal laser scanning microscopy
YFP: yellow fluorescent protein
HPI: hours post infection
DPI: days post infection
PBS: phosphate buffered saline
LB: Luria-Bertani
SOCS: suppressors of cytokine signaling
HPF: hours post fertilization.
