# Supplementary material for: Dual Transcriptomics of Host-Pathogen Interaction of Cystic Fibrosis Isolate Pseudomonas aeruginosa PASS1 With Zebrafish
Source: Front Cell Infect Microbiol. 2018 Nov 22;8:406. doi: 10.3389/fcimb.2018.00406 (PMC6262203; doi:10.3389/fcimb.2018.00406)
Supplement: Supplementary file 7 [file Data_Sheet_1.docx]

**
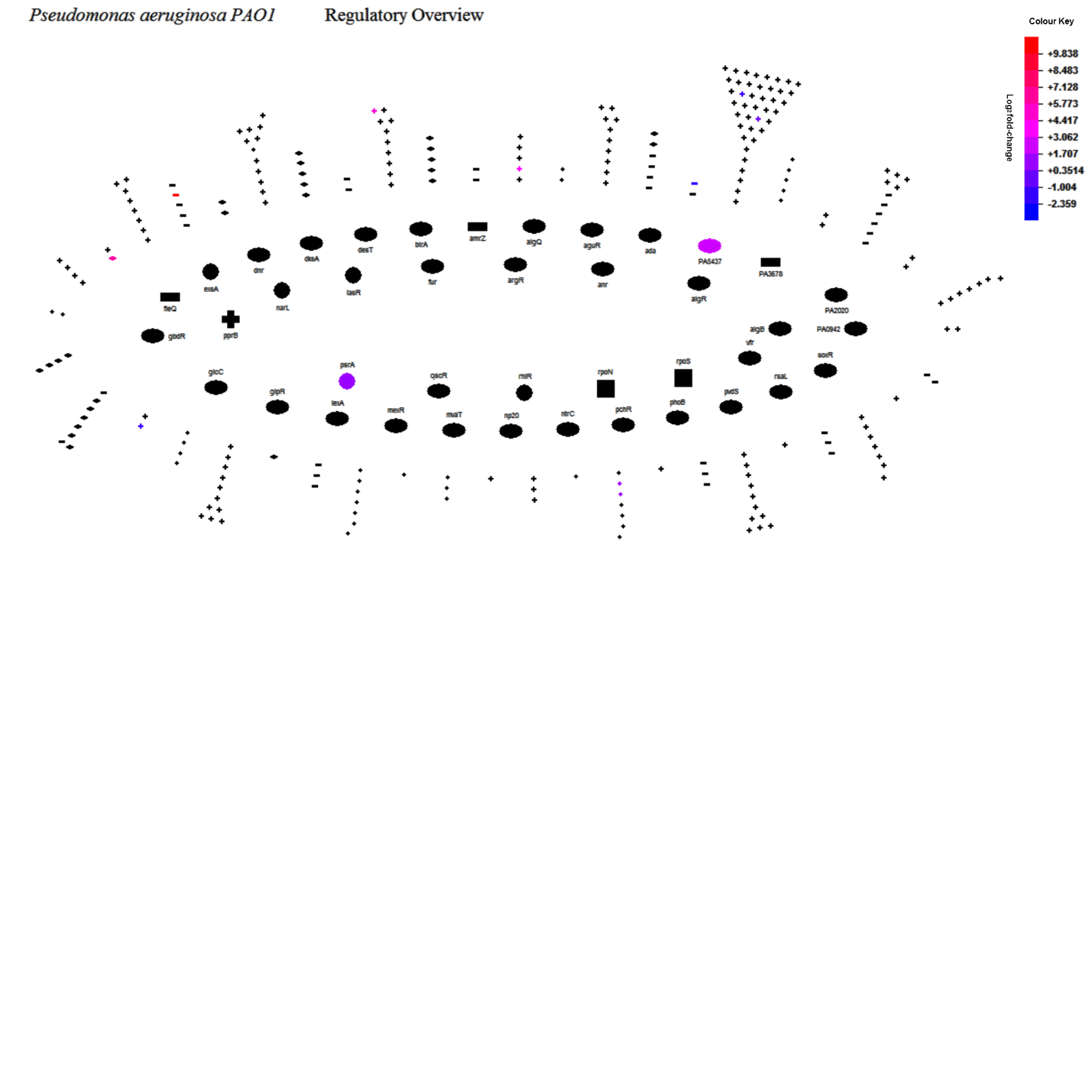
**

**PA5437**

***psrA***

**Fig S1. Regulatory overview of *P*. aeruginosa PASS1 grown *in vivo* compared to PASS1 grown in Luria-Bertani medium.** Genes with the *p ≤* 0.01, with log_2_ of fold-change of -1≥ to ≤1 cut-off were overlaid on the *P. aeruginosa* PAO1 regulatory overview in biocyc (http://www.biocyc.org). The inner ring consists of master regulators and sigma factors, the outer ring consists of genes that are regulated only, and the middle ring consists of all other genes. The color scale represents the log_2_ fold-change expression of the gene. Genes remain black in color if the dataset does not consist of a gene expression value for the particular gene.


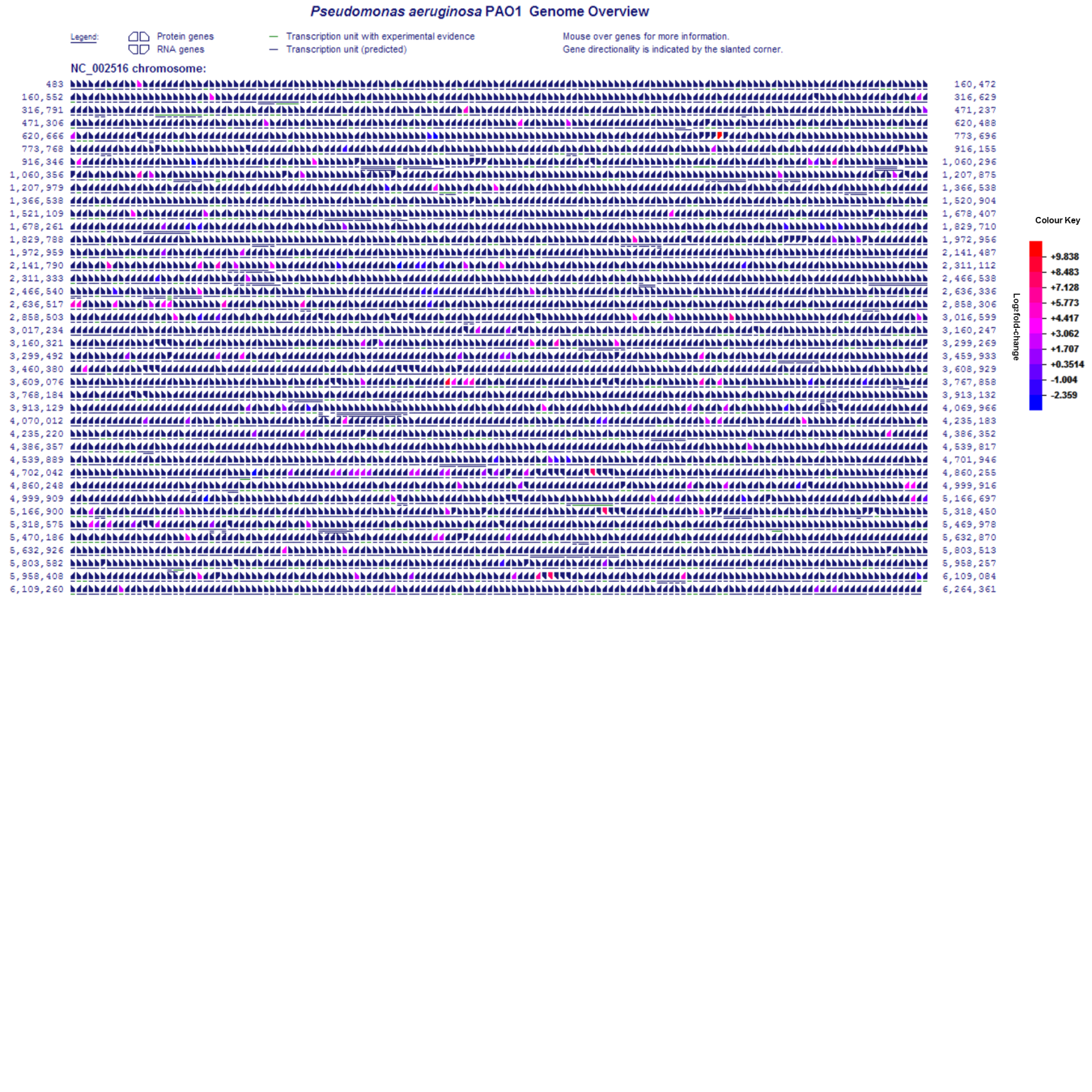
**Fig. S2. Genome overview of *P.* aeruginosa PASS1 grown in zebrafish compared to growth in Luria-Bertani medium.** Genes with the p-value of 0.01 and log_2_ fold-change -1≥ to ≤1 cut-off were overlaid on the *P. aeruginosa* PAO1 genome overview in biocyc (http://www.biocyc.org). The scaffold of the PAO1 genome displays the position of the genes within the genome. The color scale represents the log_2_ fold-change expression of the gene. Genes remain navy blue in color if the dataset does not consist of a gene expression value for the particular gene.

**
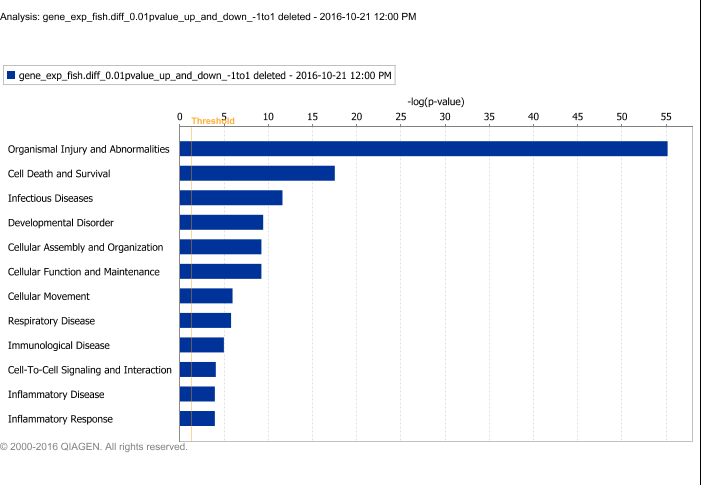
**

**Fig. S3. Molecular and cellular functions in the zebrafish embryo infected with PASS1 versus zebrafish mock-infected with phosphate buffered saline.** The bar graph image obtained via the IPA ingenuity pathway analysis system showing a total of 3238 differentially expressed (*p ≤* 0.01, log_2_ fold-change of -1≥ to ≤1 cut-off) annotated genes (S6 Table) assigned to molecular and cellular functions.


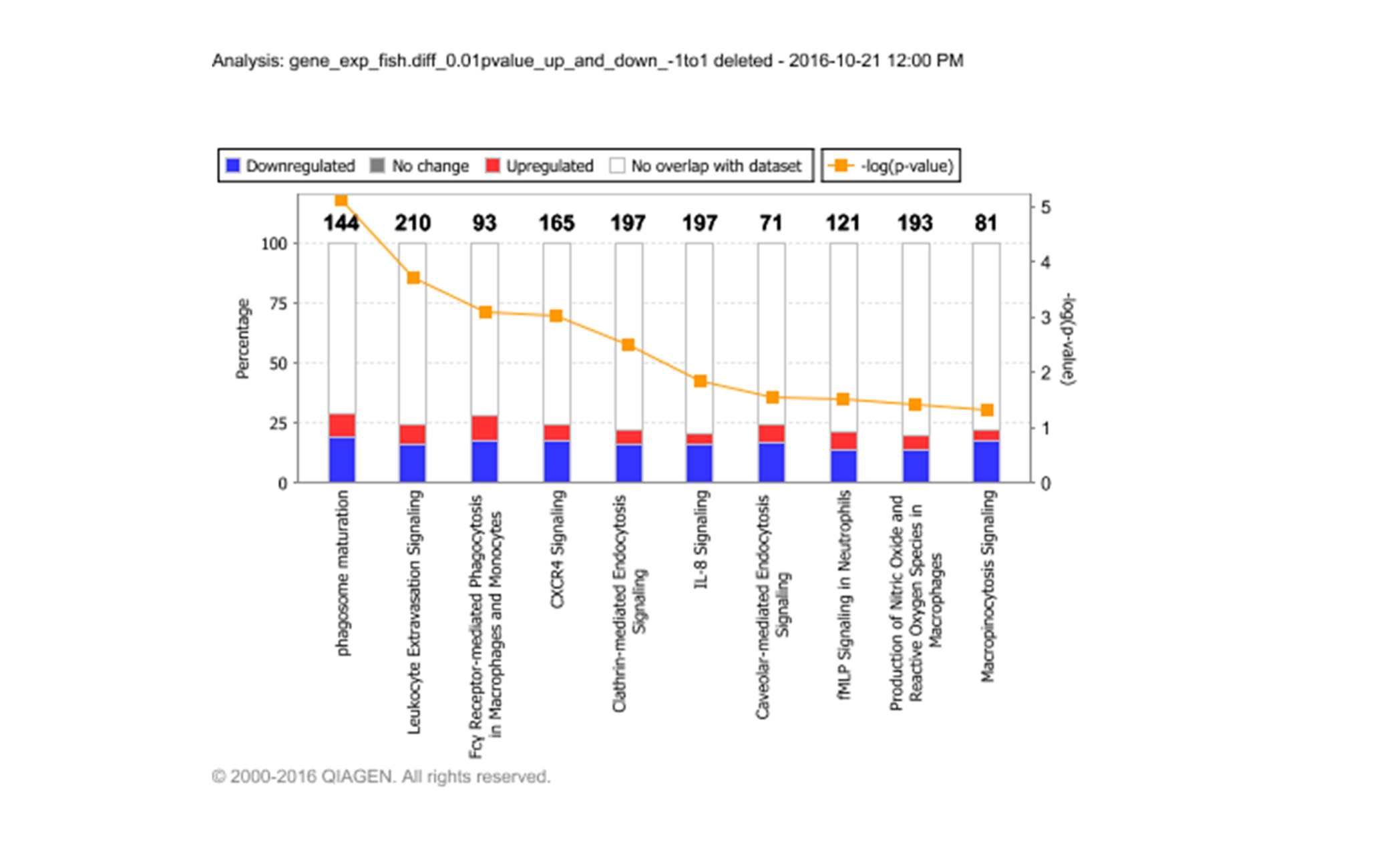


**Fig. S4. Cellular and humoral innate immune response signaling pathways enriched in zebrafish infected with PASS1.** The data was obtained via the Ingenuity Pathway Analysis (IPA) of canonical pathways significantly enriched in cellular and humoral innate immune response signaling detected by transcriptomics analysis (*p ≤* 0.01 and log_2_ fold-changes cut-off -1≥ to ≤1).


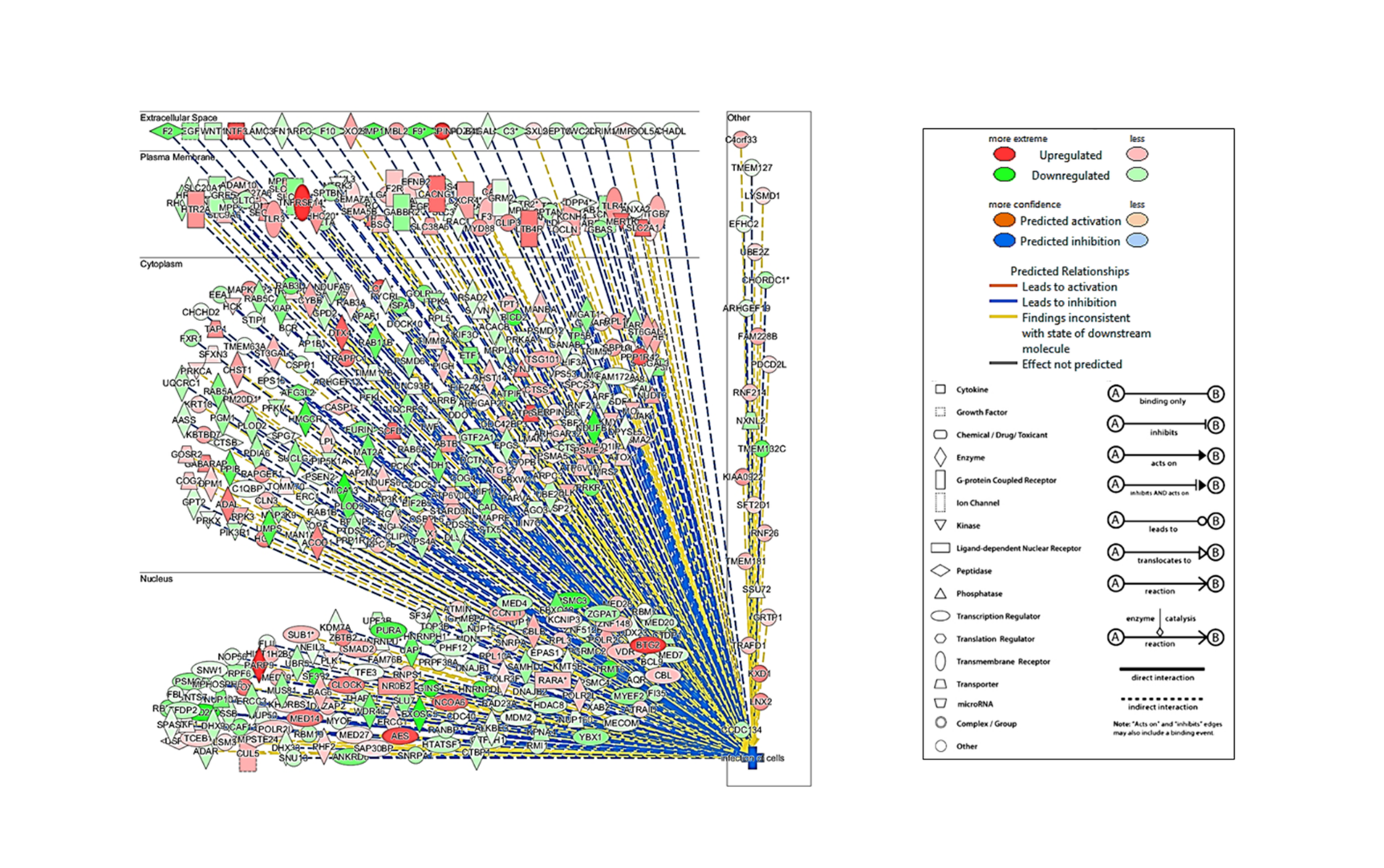


**Fig. S5. Network of genes related to infection of cells as revealed via IPA analysis of differentially expressed genes in zebrafish embryo infected with *P. aeruginosa* PASS1.** During infection of zebrafish cells with *P. aeruginosa* PASS1 various genes with varying functions were expressed in the nucleus, cytoplasm, cytoplasm and extracellular space*.* Significantly upregulated genes are shown in red and downregulated genes shown are in green with the intensity of the color showing the level of upregulated and downregulation of the gene expressed, as log2 of fold-change (*p ≤* 0.01 and log_2_ fold-changes cut-off -1≥ to ≤1).
